# Fecal Immunochemical Tests for Colorectal Cancer Screening: Is Fecal Sampling from Multiple Sites Necessary?

**DOI:** 10.3390/cancers11030400

**Published:** 2019-03-21

**Authors:** Efrat L. Amitay, Anton Gies, Korbinian Weigl, Hermann Brenner

**Affiliations:** 1 Division of Clinical Epidemiology and Aging Research, German Cancer Research Center (DKFZ), 69120 Heidelberg, Germany; k.weigl@dkfz-heidelberg.de (K.W.); h.brenner@Dkfz-Heidelberg.de (H.B.); 2 Division of Preventive Oncology, German Cancer Research Center (DKFZ) and National Center for Tumor Diseases (NCT), 69120 Heidelberg, Germany; anton.gies@nct-heidelberg.de; 3 Heidelberg Medical Faculty, Heidelberg University, 69120 Heidelberg, Germany; 4 German Cancer Consortium (DKTK), German Cancer Research Centre (DKFZ), 69120 Heidelberg, Germany

**Keywords:** colorectal neoplasms, early detection of cancer, fecal immunochemical tests, fecal sample

## Abstract

Fecal immunochemical tests (FITs) for hemoglobin (Hb) are increasingly used for colorectal cancer (CRC) screening. Most FIT manufacturers instruct that fecal samples from multiple parts of one bowel movement should be obtained. Our aim was to compare the FIT diagnostic performance based on fecal samples from just one versus two different sites of one bowel movement. A total of 1141 participants of screening colonoscopy provided two fecal samples from two different sites of a single bowel movement for FIT analyses. There was no statistically significant difference in the diagnostic performance of the FIT when either one or both fecal samples were used for analysis, with area under the curve (AUC) for detecting CRC ranging from 0.94 (95% confidence interval (CI) 0.84–0.99) for one FIT to 0.95 (95%CI 0.86–0.99) for a geometric mean of two FITs. The manufacturers’ recommendation of sampling multiple sites of the stool aims to reduce intra-individual Hb variability and improve diagnostic performance. If no such improvement can be achieved, the recommendation for multiple-site sampling might have potential adverse effects on population adherence to FIT-based CRC screening. Our results point to a potential of increasing adherence to FIT screening by simplifying instructions for fecal sampling at no loss of the diagnostic performance.

## 1. Introduction

Fecal immunochemical tests (FITs) for hemoglobin (Hb) are increasingly recommended and used for colorectal cancer (CRC) screening [1,2,3]. Typically, fecal sampling from only one bowel movement is required for FITs, as previous studies have shown little, if any, gain in diagnostic performance when combining FIT results from two or three bowel movements [4]. However, most FIT manufacturers provide detailed and not too appealing instructions on how to obtain fecal samples from multiple parts of the same bowel movement in order to account for different fecal Hb concentrations within a single bowel movement. Table 1 provides examples of stool sample instructions for a number of quantitative FITs.

However, it is unclear whether multiple-site sampling is superior to single-site fecal sampling. To the best of our knowledge, no previous study has assessed the potential gain in diagnostic performance of single-site versus multi-site fecal sampling from the same bowel movement among average-risk screening colonoscopy participants. In particular, none of the studies evaluating the diagnostic performance of FIT in a screening setting identified in a systematic review published in December 2017 (covering literature up to July 2017) reported such results [5]. 

In this study, we aimed to provide empirical evidence on inter-site variation of fecal Hb concentrations within a single bowel movement and its potential relevance for the diagnostic performance of FIT comparing single-site versus multiple-site fecal sampling in a large screening study from Germany.

## 2. Results

A total of 1141 CRC screening participants from the BliTz study were included in this analysis. Of those, 50.3% were women, and the median age was 60 years (Table 2). One hundred and twenty five participants were diagnosed with advanced neoplasms, including participants diagnosed with CRC (*n* = 14) or advanced adenoma (*n* = 111), while 1016 participants had no advanced neoplasm detected at screening colonoscopy. 

### Detecting CRC by Single-Site Versus Multiple-Site Fecal Sampling from the Same Bowel Movement

Indicators for the diagnostic performance of both FITs are shown in Table 3. There was no statistically significant difference in the test performance of the first single-site sample (FIT1) and the second single-site sample (FIT2). The sensitivity for detecting CRC was 92.9% (95% CI 66.1–99.8) for each single-site FIT and for all combinations of the two FITs (multiple-site). For detecting advanced adenomas, the sensitivity was 38.7% (95% CI 29.6–48.5) for FIT1 and 37.8% (95%CI 28.8–47.5) for FIT2. When combining CRC and advanced adenomas into a group of advanced neoplasms, the sensitivity was 44.8% (95% CI 35.9–54.0) for FIT1 and 44.0% (95% CI 35.1–53.2) for FIT2. The specificity for no advanced neoplasms was 90.0% and 89.5% for FIT1 and FIT2, respectively.

Combining FIT results according to algorithm I (PP-two positive FIT results) resulted in a lower sensitivity of 38.4% (95% CI: 29.8–47.5) for the detection of advanced neoplasms, and a higher specificity of 93.3% (95% CI: 91.6–94.8) for no advanced neoplasms. When combining both FIT results according to algorithm II (PN-at least one positive FIT result), the sensitivity for detecting advanced neoplasms increased to 50.4% (95% CI 41.3–59.5), with the specificity decreasing to 86.1% (95%CI 83.8–88.2). Combinations based on the arithmetic or geometric mean, a simulation for multi-site sampling of the stool, resulted in sensitivities and specificities that were similar to those of the single FITs for detecting CRC, advanced adenomas, or their combination. 

**Figure 1:**
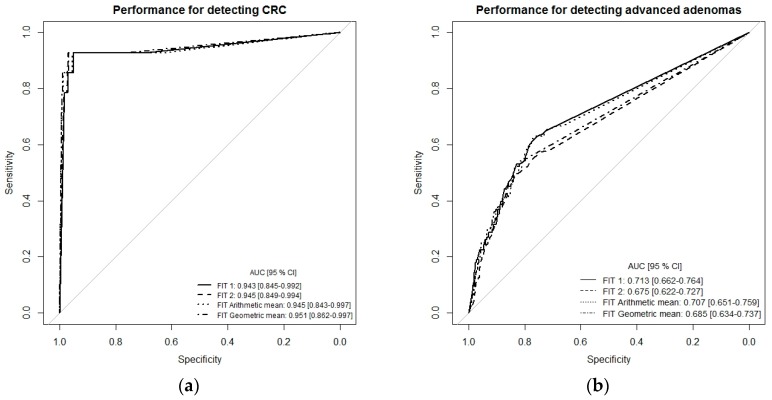

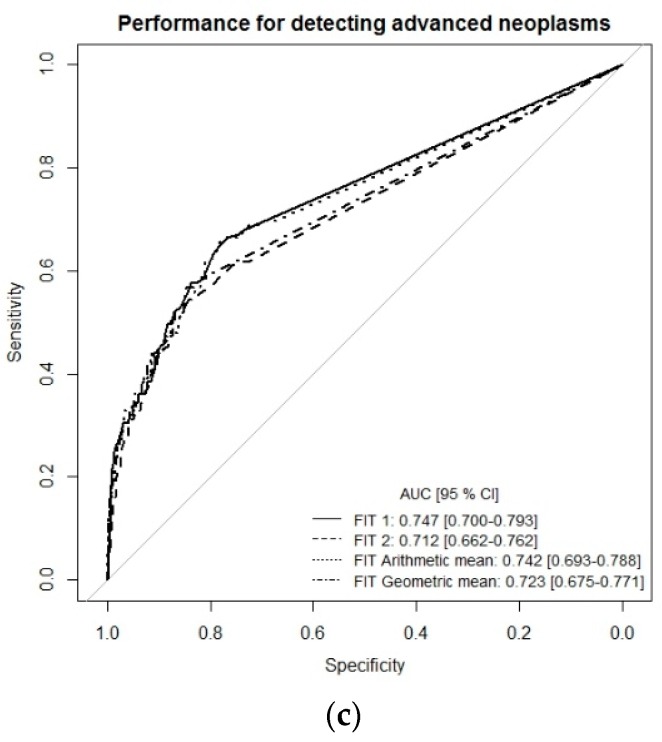
Receiver-operating characteristic (ROC) curve analysis comparing diagnostic performance of FITs for single-site versus multiple-site fecal sampling from the same bowel movement. (**a**) *n* = 14 CRC cases, (**b**) *n* = 111 Advanced adenomas, (**c**) *n* = 125 advanced neoplasms. Abbreviations: FIT, fecal immunochemical test; CRC, colorectal cancer; AUC, area under the curve.

To get a more comprehensive picture of the diagnostic performance of the single-site FITs (FIT1 or FIT2) and the combination of both FITs (multiple-site, simulated by arithmetic and geometric means), we performed receiver-operating characteristic (ROC) curve analysis (Figure 1a–c). For the detection of CRC, ROC curves and areas under the curves (AUCs) were very similar whether using the results of each FIT separately (single-site) or a combination of both tests (multiple-site), with AUCs ranging from 0.943 (95% CI 0.845–0.992) to 0.951 (95% CI 0.862–0.997). The AUC for detecting advanced adenomas ranged between 0.676 (95% CI 0.662–0.727) for FIT2 and 0.685 (95% CI 0.634–0.737) for FIT geometric mean. For the combined endpoint advanced neoplasm, AUCs were 0.747 (95% CI 0.700–0.793) for FIT1, 0.712 (95% CI 0.662–0.762) for FIT2, 0.742 (95% CI 0.693–0.788) for the arithmetic mean of both FITs, and 0.723 (95% CI 0.675–0.771) for the geometric mean of both FITs (Figure 1c).

Spearman’s rank correlation between the two FITs (single-site) was 0.731. For participants with no detectable blood in FIT1 (*n* = 787), 98% had Hb concentrations below the manufacturer’s cutoff (17 µg Hb/g stool) also in FIT2. For participants with detectable Hb concentrations below the manufacturer’s cutoff in FIT1 (*n* = 196), 15% had Hb concentrations above the manufacturer’s cutoff in FIT2.

## 3. Discussion

With FIT increasingly being used for detecting advanced adenomas and CRC in screening programs worldwide [2], we looked at providing empirical evidence on inter-site fecal Hb variation within the same bowel movement. To our knowledge, this is the first study evaluating and directly comparing the diagnostic performance of single-site versus multiple-site fecal sampling of the same bowel movement in screening colonoscopy participants. We observed a similar diagnostic performance between single-site and multiple-site fecal sampling for detecting both CRC and advanced adenoma.

In the presence of the major heterogeneity of Hb concentrations within a single bowel movement, FIT manufacturers, in the leaflets for patients accompanying the stool collection tubes, call for sampling the stool at multiple sites of the bowel movement (Table 1); the rationale behind this recommendation being that multiple-site sampling may reduce intra-individual variability of FIT results and improve the diagnostic performance. On the other hand, if no such improvement can be achieved, the recommendation for multiple-site sampling might be irrelevant or even harmful as unnecessarily complex or unpleasant fecal sampling schemes might have potential adverse effects on population adherence to FIT-based CRC screening [6]. 

Although our results indicate that fecal Hb concentrations can differ slightly between different sites of the same bowel movement, combining both FIT results by calculating either an arithmetic or geometric mean, which may simulate stool sampling from multiple sites of the stool sample as is recommended by FIT manufacturers, did not improve the test performance for detecting CRC or advanced adenomas. Similarly, the ROC curves and AUCs were almost identical for single-site versus multiple-site sampling, indicating that multiple-site sampling did not improve the overall diagnostic performance across a wide range of cutoffs compared to single-site fecal sampling. 

A few previous colonoscopy-controlled studies [7,8] compared the diagnostic performance of FITs in average-risk screening populations with regards to the number of FIT samples. The authors found that with an increasing number of FIT samples, the sensitivity increases too, but in a similar way, the specificity decreases. However, looking at the overall test performance, similar AUCs for the detection of advanced neoplasms were observed. Since the samples for these studies were taken on consecutive days from different bowel movements, the question of whether multiple-site fecal sampling improves the overall test performance of FIT compared to single-site sampling from the same bowel movement was not directly addressed.

The meta-analysis by Lee et al [4], published in 2014, also looked at aspects of the diagnostic accuracy of one, two, or three samples for FITs taken from consecutive bowel movements for the detection of CRC in average-risk screening populations. The authors concluded that the characteristics of FIT, such as sensitivity, specificity, positive likelihood ratio, and negative likelihood ratio, were very similar, irrespective of the number of stool samples tested, although the authors found significant heterogeneity in the sensitivity and specificity rates between studies. 

The strengths of our study lie in its setting within a true screening population. All the participants in our study, not only those with a positive FIT result, underwent screening colonoscopy, independent of the FIT result, thus enabling us to have a comprehensive look at the diagnostic characteristics of the FIT. Moreover, to our knowledge, this is the first study to compare two stool samples taken on the same day, and from different areas of the same bowel movement. A limitation of our study is the fact that stool samples were not collected directly in original FIT sampling tubes, which are filled with a preservative buffer to slow down hemoglobin decay, but in small containers, and stored frozen until analysis. However, we have previously shown that collection in small containers or samples collected by the participants in FIT sampling tubes provided very comparable data. A comparison of frozen and fresh fecal samples also provided similar results [9]. Furthermore, the analysis was based on fecal sampling from only two different sites of the same bowel movement, while some manufacturers recommend sampling of up to six places in the same stool.

## 4. Materials and Methods

### 4.1. Study Design and Population

Our analysis is based on data from the ongoing BliTz study, whose design has been reported in detail elsewhere [10,11,12,13]. Briefly, the BliTz study was initiated in 2005 with the aim of evaluating and improving non-invasive tools for CRC screening and includes participants of the German screening colonoscopy program, recruited in 20 gastroenterological practices in southwestern Germany. Written informed consent was obtained from each participant in the study. Participants were given stool collection containers for collecting fecal samples prior to preparation for colonoscopy and asked to fill out a self-administered questionnaire including questions regarding health history and lifestyle. Colonoscopy and histology results were collected for all participants. The study protocol conforms to the ethical guidelines of the Declaration of Helsinki as reflected by the approval by the ethics committees of the Medical Faculty of Heidelberg University (178/2005) and those of the state chambers of physicians of Baden-Württemberg (M118-05-f), Rhineland-Palatine (837.047.06(5145)) and Saarland (217/13). The BliTz study was registered in the German Clinical Trials Register (DRKS-ID: DRKS00008737). 

**Figure 2:**
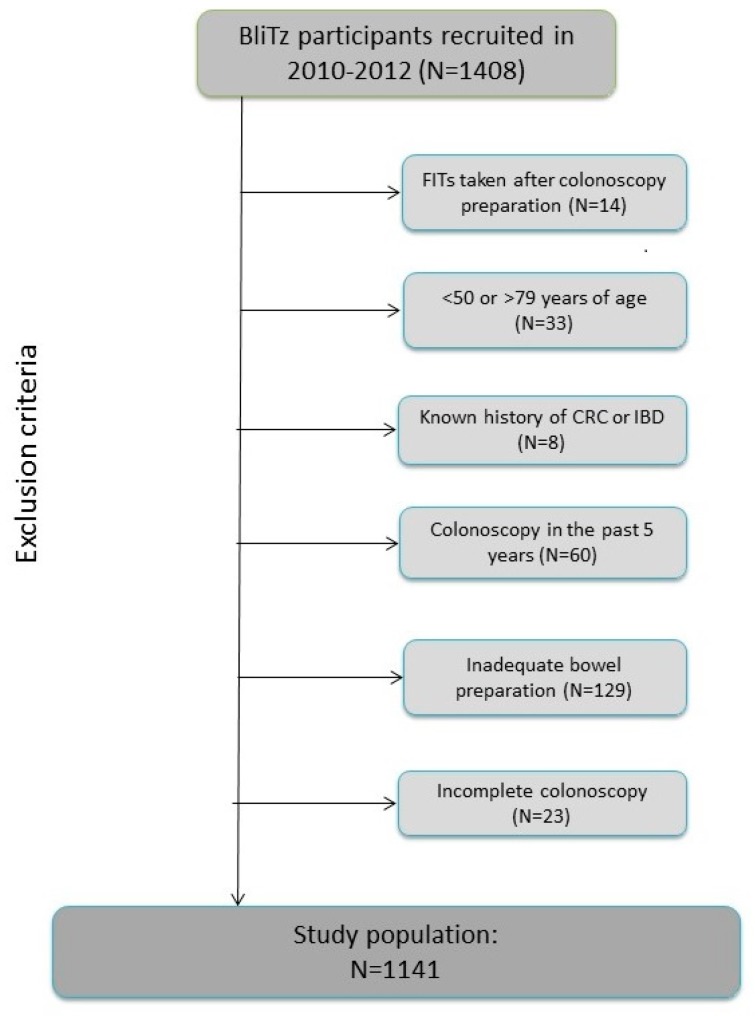
Study inclusion criteria. Abbreviations: CRC, colorectal cancer; IBD, inflammatory bowel disease.

The current analyses include BliTz study participants recruited between 2010 and 2012 and the following were excluded from the current analyses (Figure 2): participants who conducted stool sampling after preparation for colonoscopy or after colonoscopy (*n* = 14), those under 50 years or over 79 years of age at the time of colonoscopy (*n* = 33), participants who reported that they had been diagnosed with CRC in the past or who were suffering from inflammatory bowel disease (*n* = 8), those who had undergone another colonoscopy in the five years prior to the current colonoscopy (*n* = 60), and participants who had inadequate bowel preparation prior to colonoscopy (*n* = 129) or an incomplete colonoscopy (caecum not reached) (*n* = 23). In total, 1141 participants met all inclusion criteria and were included in this study. 

### 4.2. Data and Sample Collection

After signing informed consent forms, BliTz study participants were given two small stool collection containers. They were instructed to collect one stool sample per container, with each sample from a different area of the same bowel movement. Stool collection was done at home before bowel preparation for colonoscopy. No dietary or medicinal recommendations or restrictions were given. The participants were asked to keep the samples frozen, or refrigerated if freezing was not possible, and to bring them to the gastroenterological practice on the day of their colonoscopy. The samples were then directly stored at −20 °C and shipped on dry ice to a central laboratory (see below). Demographic information was obtained from the self-administered questionnaires filled out by all participants.

### 4.3. FIT Analyses

FIT analyses using FOB Gold by Sentinel Diagnostics (Milan, Italy) were evaluated blinded at a central DIN EN ISO 15189 accredited laboratory (MVZ Labor Limbach, Heidelberg, Germany). Reporting and evaluation of the FITs followed FITTER standards [14]. Each collection container held 1 g of stool and the median time from collection to analysis was five days (IQR = 4–7 days). In the lab, the frozen stool samples were thawed and an automatic stool extraction system was used to extract 10 mg stool, which was then diluted in 1.7 mL extraction buffer (i.e., dilution: 1:170) according to routine clinical practice. The samples were assigned as FIT1 or FIT2 by simple randomization. Both samples were analyzed using Abbott Architect c8000 (Abbott Park, IL, USA) with an analytical working range of 0.034–140 µg Hb/g stool on the same date, which was recorded. Classification of FIT results as positive or negative was done at the threshold recommended by the manufacturer (17 µg Hb/g stool).

### 4.4. Statistical Analysis

The current analysis is a post hoc analysis of a sub-group in a large diagnostic study designed to estimate the diagnostic performance of various non-invasive tests compared to screening colonoscopy. This study was therefore not specifically powered or designed to test a specific pre-defined hypothesis. All statistical analyses were conducted using R version 3.4.4 (2018-03-15) [15]. The positivity rate, sensitivity for the detection of CRC, advanced adenomas (defined as adenomas with at least one of the following: ≥1 cm in size, tubulovillous or villous components and high-grade dysplasia) or their combination (advanced neoplasms), as well as specificity for the absence of advanced neoplasms with their exact 95% confidence intervals (CIs), were calculated for each FIT separately and in combination. For the combination of both FIT results, four different algorithms were applied: (1) Positive if at least one of the FIT results was above the manufacturer’s cutoff; (2) positive if both FIT results were above the manufacturer’s cutoff; (3) positive if the arithmetic mean of the results of the two FITs was above the manufacturer’s cutoff; and (4) positive if the geometric mean of the results of the two FITs was above the manufacturer’s cutoff. Indicators of diagnostic performance were compared using McNemar’s exact test.

Spearman’s rank correlation was used to assess the correlation between the two quantitative FIT results. In order to evaluate the diagnostic performance across different cutoffs, receiver operating characteristic (ROC) curves were plotted and the areas under the curve (AUCs) for the detection of CRC, advanced adenomas, and both of these outcomes combined (advanced neoplasms) were determined using the “pROC” package [16] in R. Confidence intervals (95% CIs) of the AUCs were calculated via nonparametric bootstrapping, replicating random sampling with replacement. Statistical significance of two-sided tests was defined by *p*-values < 0.05.

## 5. Conclusions

In conclusion, despite its limitations, our study suggests that the diagnostic performance of FIT utilizing multiple-site fecal sampling from the same bowel movement may not be superior to a single-site sample in the average-risk screening population for the detection of CRC and advanced adenomas. These results do not support the necessity for sampling the stool in different locations for a FIT, as currently recommended by most manufacturers. Our findings suggest that the simplification of patient instructions for FITs might be considered, as the advantages of the expected increase in patient adherence to simplified instructions may outweigh the negligible, if any, loss in diagnostic performance.

## Figures and Tables

**Table 1:** Stool collection instructions for quantitative FITs from manufacturers’ consumer leaflets.

FIT Brand	Manufacturer	Stool Collection Instructions as Given by the Manufacturer	Number of Sites
OC-Sensor	Eiken Chemical co., Tokyo, Japan	Collect fecal samples by scraping the surface of the stools in different areas	Scrape the surface
MagStream HemSp	Fujirebio, Tokyo, Japan	Put the tip of the blue stick into your bowel motion and drag back and forth	Scrape the surface
HM-JACKarc	Kyowa Medex, Tokyo, Japan	Scrape the end of the stick along the stool sample	Scrape the surface
RIDASCREEN hemoglobin	R-Biopharm, Darmstadt, Germany	Swab the stool with the sampling rod	Swab the surface
SENTiFIT FOB Gold	Sentinel Diagnostics, Milan, Italy	Insert the stick into 4 different places, scraping the surface in a crosswise motion	4 + scrape the surface
Eurolyser FOB test	Eurolyser Diagnostica, Salzburg, Austria	Insert the stick into the stool sample at 5 to 6 different positions to ensure a homogenous sampling	5–6
FIT NS-Prime	Alfesa Pharma, Osaka, Japan	Collect sample feces onto the gutters of the collector stick by scraping 4–5 areas on the surface of fecal specimens	4–5
QuantOn Hem	Immundiagnostik, Bensheim, Germany	Prick the tip of the sample collection stick in at least 3 different locations in the stool sample in one go	3+
QuikRead go iFOBT	Orion Diagnostica, Espoo, Finland	Collect the sample by poking the sample collecting stick… in three (3) different places on the faeces sample	3
IDK Hemoglobin ELISA	Immundiagnostik, Bensheim, Germany	“Insert the dosing stick 3-times into different areas of the stool specimen	3
ImmoCARE-C	CARE diagnostic, Voerde, Germany	Insert the applicator stick upright into the stool at 3 different areas in one go	3

FIT: fecal immunochemical test.

**Table 2:** Characteristics of the study population.

Characteristic		*n* = 1141	%
Sex	Female	574	50.3
	Male	567	49.7
Age (years)	50–54	32	2.8
	55–59	514	45.0
	60–64	240	21.0
	65–69	170	14.9
	70–74	131	11.5
	75–79	54	4.7
	median	60	
Most advanced finding at screening colonoscopy	**Any advanced neoplasm**	**125**	**11.0**
	Colorectal cancer	14	1.2
	Advanced adenoma	111	9.7
	**No advanced neoplasm**	**1016**	**89.0**
	Non-advanced adenoma	236	20.7
	None of above *	780	68.4

* None of above = Hyperplastic polyp (*n* = 110); Non defined polyps (*n* = 8); Other non-malignant diagnosis (*n* = 31); No findings (*n* = 631).

**Table 3:** Indicators of test performance for detecting advanced neoplasms by single-site vs multiple-site fecal sampling from one bowel movement.

Indicator of Test Performance at Cutoff of 17 µg Hb/g Stool		FIT					
		Single-Site		Multiple-Site			
		FIT1	FIT2	PP	PN	A_Mean	G_Mean
Sensitivity for CRC (*n* = 14)	*n*	13/14	13/14	13/14	13/14	13/14	13/14
	%	92.9	92.9	92.9	92.9	92.9	92.9
	95% CI	66.1–99.8	66.1–99.8	66.1–99.8	66.1–99.8	66.1–99.8	66.1–99.8
	*p*-value ^+^	Reference	1	1	1	1	1
		1	Reference	1	1	1	1
Sensitivity for advanced adenomas (*n* = 111)	*N*	43/111	42/111	35/111	50/111	43/111	40/111
	%	38.7	37.8	31.5	45.0	38.7	36.0
	95% CI	29.6–48.5	28.8–47.5	23.0–41.0	35.6–54.8	29.6–48.5	27.1–45.7
	*p*-value ^+^	Reference	1	0.008	0.016	1	0.453
		1	Reference	0.016	0.008	1	0.727
Sensitivity for any advanced neoplasms (*n* = 125)	*N*	56/125	55/125	48/125	63/125	56/125	53/125
	%	44.8	44.0	38.4	50.4	44.8	42.4
	95% CI	35.9–54.0	35.1–53.2	29.8–47.5	41.3–59.5	35.9–54.0	33.6–51.6
	*p*-value ^+^	Reference	1	0.008	0.016	1	0.453
		1	Reference	0.016	0.008	1	0.727
Specificity for no advanced neoplasms (*n* = 1016)	*N*	914/1016	909/1016	948/1016	875/1016	904/1016	931/1016
	%	90.0	89.5	93.3	86.1	89	91.6
	95% CI	87.9–91.7	87.4–91.3	91.6–94.8	83.8–88.2	86.9–90.8	89.8–93.3
	*p*-value ^+^	Reference	0.640	<0.001	<0.001	0.110	0.008
		0.640	Reference	<0.001	<0.001	0.533	<0.001

CI, confidence interval; CRC, colorectal cancer; FIT, fecal immunochemical test; Hb, hemoglobin. FIT result definitions: FIT1: Positive if FIT1 is above cutoff; FIT2: Positive if FIT2 is above cutoff; PP: positive if both FIT1 and FIT2 are above cutoff; PN: Positive if at least one of both tests are above cutoff; A_Mean: Positive if the arithmetic mean of both FITs is above cutoff; G_Mean: Positive if the geometric mean of both FITs is above cutoff. + McNemar exact test.

## References

[1] Halloran S.P., Launoy G., Zappa M. (2012). International Agency for Research on C. European guidelines for quality assurance in colorectal cancer screening and diagnosis. First Edition—Faecal occult blood testing. Endoscopy.

[2] Schreuders E.H., Ruco A., Rabeneck L. (2015). Colorectal cancer screening: A global overview of existing programmes. Gut.

[3] Bibbins-Domingo K., Grossman D.C., USPSTF (2016). Screening for Colorectal Cancer: US Preventive Services Task Force Recommendation Statement. JAMA.

[4] Lee J.K., Liles E.G., Bent S., Levin T.R., Corley D.A. (2014). Accuracy of fecal immunochemical tests for colorectal cancer: Systematic review and meta-analysis. Ann. Intern. Med..

[5] Gies A., Bhardwaj M., Stock C., Schrotz-King P., Brenner H. (2018). Quantitative fecal immunochemical tests for colorectal cancer screening. Int. J. Cancer.

[6] Von Wagner C., Good A., Smith S.G., Wardle J. (2012). Responses to procedural information about colorectal cancer screening using faecal occult blood testing: The role of consideration of future consequences. Health Expect..

[7] Hernandez V., Cubiella J., Gonzalez-Mao M.C. (2014). Fecal immunochemical test accuracy in average-risk colorectal cancer screening. World J. Gastroenterol..

[8] Park D.I., Ryu S., Kim Y.H. (2010). Comparison of guaiac-based and quantitative immunochemical fecal occult blood testing in a population at average risk undergoing colorectal cancer screening. Am. J. Gastroenterol..

[9] Chen H., Werner S., Brenner H. (2017). Fresh vs Frozen Samples and Ambient Temperature Have Little Effect on Detection of Colorectal Cancer or Adenomas by a Fecal Immunochemical Test in a Colorectal Cancer Screening Cohort in Germany. Clin. Gastroenterol. Hepatol..

[10] Hundt S., Haug U., Brenner H. (2009). Comparative evaluation of immunochemical fecal occult blood tests for colorectal adenoma detection. Ann. Intern. Med..

[11] Brenner H., Tao S., Haug U. (2010). Low-dose aspirin use and performance of immunochemical fecal occult blood tests. JAMA.

[12] Brenner H., Tao S. (2013). Superior diagnostic performance of faecal immunochemical tests for haemoglobin in a head-to-head comparison with guaiac based faecal occult blood test among 2235 participants of screening colonoscopy. Eur. J. Cancer.

[13] Werner S., Krause F., Rolny V. (2016). Evaluation of a 5-Marker Blood Test for Colorectal Cancer Early Detection in a Colorectal Cancer Screening Setting. Clin. Cancer Res..

[14] Fraser C.G., Allison J.E., Young G.P., Halloran S.P., Seaman H. (2014). A standard for Faecal Immunochemical TesTs for haemoglobin evaluation reporting (FITTER). Ann. Clin. Biochem..

[15] R Core Team (2018). R: A Language and Environment for Statistical Computing.

[16] Robin X., Turck N., Hainard A. (2011). pROC: An open-source package for R and S+ to analyze and compare ROC curves. BMC Bioinform..

